# Pathogenic role of PFKFB3 in endothelial inflammatory diseases

**DOI:** 10.3389/fmolb.2024.1454456

**Published:** 2024-09-10

**Authors:** Ling Zhou, Juan Li, Juanjuan Wang, Xuping Niu, Junqin Li, Kaiming Zhang

**Affiliations:** ShanXi Key Laboratory of Stem Cells for Immunological Dermatosis, State Key Breeding Laboratory of Stem Cells for Immunological Dermatosis, Taiyuan Central Hospital, Taiyuan, China

**Keywords:** PFKFB3, endothelial cells, glycolysis, inflammatory, 3PO

## Abstract

The differentiation of vascular endothelial cells and the formation of new blood vessels are inseparable from the energy supply and regulation of metabolism. The budding of blood vessels is a starting point of glycolysis pathway in angiogenesis. Phosphofructokinase-2/fructose 2,6-biophosphatase 3 (PFKFB3), a key rate-limiting enzyme in glycolysis, exhibits strong kinase activity. Inhibition of PFKFB3 can reduce the rate of glycolysis, thereby inhibiting the budding of blood vessels, resulting in inhibition of pathological angiogenesis. In this review, the role of PFKFB3 in the angiogenesis of inflammatory diseases was summarized, and the endothelial inflammatory diseases associated with PFKFB3 were reviewed.

## Introduction

**FIGURE 1:**
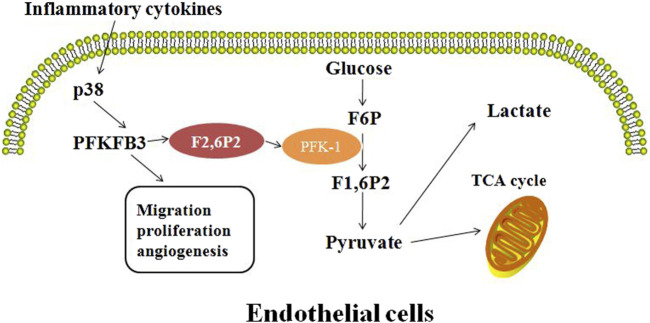
Inflammation affects the angiogenic profile of endothelial cells by upregulation of PFKFB3 expression and regulation of fructose-6-phosphate substrate cycle.

Glycolysis is a metabolic pathway that converts glucose into pyruvate (Ganapathy-Kanniappan and Geschwind, 2013). Free energy released during this process is used to form the high-energy compounds, ATP and NADH (Luengo et al., 2021). Cancer cells tend to rely on glycolysis rather than oxidative phosphorylation, even when oxygen supplies are plentiful. This phenomenon is known as the “Warburg effect” (Vaupel et al., 2019; Kang et al., 2023). The rate of glycolysis flux is controlled by various mechanisms at different levels. One of the rate-limiting checkpoints for glycolytic flux is the conversion of fructose-6-phosphate (F6P) to fructose-1, 6-diphosphate (F1, 6P2) by fructose-6-phosphate kinase (PFK-1) (Kim et al., 2017; Chesney et al., 2005; Kanai et al., 2019a; Kanai et al., 2019b; Atsumi et al., 2005). The intracellular allosteric regulator, fructose 2, 6-bisphosphate (F2, 6P2), is an effective activator of PFK-1 (Atsumi et al., 2005; Cordero-Espinoza and Hagen, 2013; Hue and Rider, 1987; Pilkis et al., 1990; Minchenko et al., 2003). F2, 6P2 increases the affinity of PFK-1 for F6P and overcomes the steric inhibition of ATP on PFK-1, allowing glycolysis flux to pass through the PFK-1 checkpoint and to enter F1, 6P2 synthesis (Abuelgassim and Khoja, 1988). F2, 6P2 intracellular homeostasis is controlled by homodimers of the pfK-2/FBPase (PFKFB) family. Although the core catalytic domain of PFKFB (Ganapathy-Kanniappan and Geschwind, 2013; Luengo et al., 2021; Vaupel et al., 2019; Kang et al., 2023) has high sequence homology (85%), their four isoenzymes display different characteristics, including tissue expression profile, kinase/phosphatase activity ratio, and their response to protein kinase, hormone, and growth factor. The relationships among them is shown in Figure 1 (Minchenko et al., 2003; Okar et al., 2001; Heydasch et al., 2021).

The *Pfkfb3* gene was first cloned from fetal brain cDNA libraries, and PFKFB3 protein is widely expressed in all tissues, especially in proliferating tissues, transformed cells, solid tumors and leukemia cells (Long et al., 2019; Jiang et al., 2022). The bifunctional enzyme encoded by the *Pfkfb3* gene has the highest kinase-phosphatase activity ratio (710 times), thereby controlling the rate of glycolysis by maintaining intracellular F2,6-P2 (Kotowski et al., 2021; Chesney, 2006; Calvo et al., 2006). In addition, there are different segmentation variants of PFKFB3 (Heydasch et al., 2021; Bando et al., 2005; Duran et al., 2008). So far, at least six splicing variants of PFKFB3 have been identified in the human brain (Bando et al., 2005). The activity and localization of these splicing variants may contribute to the regulation and function of PFKFB3 in glycolysis of tumor cells, as well as its requirement for tumor growth. The *Pfkfb3* gene is located on chromosome 10P15.1 (Fleischer et al., 2011) and contains multiple copies of the AUUUA unstable element in its three untranslated regions (3 UTR) (Kanai et al., 2019b). The *Pfkfb3* gene spans 109,770 bp, and contains at least 19 exons (Mahlknecht et al., 2003). The COOH terminal variable splicing results in at least six structural isomers, known as UBI2K1-6 in humans (Zscharnack et al., 2009). PFKFB3 protein is a homodimer (Min et al., 2021). The monomer structure is divided into two functional domains within the same peptide. The C-terminal domain contains the bisphosphatase activity. This domain catalyzes the hydrolysis and degradation of F2, 6P2 to F6P and inorganic phosphate. The N-terminal domain is responsible for synthesis of F2,6P2 from F6P and ATP (Kim et al., 2006).

Expression levels of PFKFB3 are upregulated during cell mitosis, inflammation and under hypoxic stimulation (Doménech et al., 2015; Yang et al., 2021; Zhang et al., 2021; Cao et al., 2019; Wang et al., 2021). Various stimuli, including hypoxia, progesterone, estrogen and stress can induce expression of the *Pfkfb3* gene (Alvarez et al., 2021; Chédeville et al., 2020; Hamilton et al., 1997; Trenti et al., 2017; Novellasdemunt et al., 2013). These factors bind to specific sequences in the PFKFB promoter, and are the consensus hypoxia response element, progesterone response element, estrogen response element, and serum response element. The *Pfkfb3* gene expression can also be upregulated by other factors, such as IL-6, lipopolysaccharides (LPS), adenosine, and different stress stimuli (NaCl, H2O2, UV radiation or anisomycin) (Zhou et al., 2022). Inflammatory cytokines and stress stimuli increase PFKFB3 production through the P38/MK2/SRF pathway. On the other hand, PFKFB3 can upregulate expression levels of insulin, IL-6, LPS, adenosine, and mitogenic lectins such as concanavin, plant lectins and transforming growth factor-β1 (TGF-β1) (Lu et al., 2020; Zou et al., 2017; Wang et al., 2019; Ruiz-García et al., 2011).

### PFKFB3 in endothelial cells

The lumen of blood vessels is lined with a single layer of endothelial cells (ECs), including tip cells and stalk cells, each with a specific function. Endothelial cells normally lie dormant in the adult body. But during normal physiological processes or upon injury, these cells can be activated rapidly to form new vascular branches (Stapor et al., 2014). The three main metabolic pathways of energy and biomass produced by endothelial cells are glucose, fatty acid and amino acid metabolism. Because EC has a relatively low mitochondrial content (Stapor et al., 2014) and is primarily dependent on glycolysis, vascular endothelial cells rarely utilize the oxidative phosphorylation metabolic pathway, even when oxygen is abundant, but instead use glycolysis for energy (Potente and Carmeliet, 2017; Vander Heiden et al., 2009), possibly because glycolysis produces ATP more quickly than the oxidative metabolic pathway to meet the energy requirements of ECs.

**FIGURE 2:**
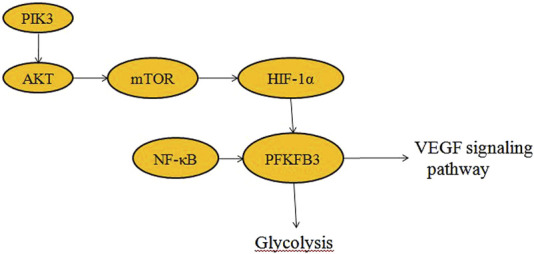
Signaling pathways associated with PFKFB3 (Akt, HIF-1α, mTOR, NF-κB and VEGF).

Glycolysis provides energy for and regulates vascular growth (De Bock et al., 2013a; Eelen et al., 2018). PFKFB3 (PFK2) is a highly expressed glycolytic enzyme in vascular ECs (Wang et al., 2019). PFKFB3 converts F6P to F2, 6P2, which is an allosteric activator of PFK-1 (Yalcin et al., 2009; Gao et al., 2020; Pegoraro et al., 2013). Therefore, inhibition of PFKFB3 enzyme can only partially inhibit the glycolytic pathway and enable cells to enter the resting phase. Angiogenesis is a dynamic process that requires the involvement of a variety of endothelial cells. The tip cells, which have many threadlike pseudopodia, act as “pioneers” during angiogenesis, first migrating to the site where the new blood vessels will grow, and then being followed by the highly proliferative stalk cells to form the new blood vessels. Vascular endothelial growth factor (VEGF) activates tip cells (Melincovici et al., 2018; Ferrara et al., 2005). Angiogenesis is the process of growing new small blood vessels from existing ones (Huang and Nan, 2019). VEGF induces PFKFB3 expression to promote angiogenesis and EC migration by regulating the formation and directional migration of tubular and lamellar feet. Silencing of PFKFB3 in ECs reduces vascular germination by reducing cell apical migration and stalk cell proliferation (Figure 2).

ECs rely more on glycolysis than oxidative phosphorylation for ATP synthesis (Eelen et al., 2015) and loss of PFKFB3 in ECs impairs blood vessel formation (De Bock et al., 2013a; Stapor et al., 2014). Inhibition of glycolysis activity suppresses invasive angiogenesis in eye diseases and inflammatory disease (Liu et al., 2024; Feng et al., 2021). Targeting PFKFB3 in ECs normalizes tumor vascularity and significantly prevents metastasis (Teuwen et al., 2017). The nuclear factor (erythrocyte derived 2) -like2 (Nrf2) regulates endodermal glycolysis and proliferation through transcriptional regulation of PFKFB3, VEGFRA, FOXO1 and MYC (Kuosmanen et al., 2018). Silencing PFKFB3 expression decreases phosphorylation of Akt (Xu et al., 2014) and disrupts CtBP1 oligomerization (Guo et al., 2024), an NADH-sensitive transcriptional co-repressor.

### PFKFB3 in inflammatory diseases

Glycolytic metabolism plays an important role in inflammation, including endothelial inflammation (Yang et al., 2021). Endothelial cells are highly glycolytic, with glycolytic rates equal to or exceeding those of some cancer cells (De Bock et al., 2013a). PFKFB3-mediated glycolysis in endothelial cells is critical in angiogenesis and the development of inflammatory diseases, such as retinopathy (Min et al., 2021), pulmonary hypertension (Cao et al., 2019), and metastatic cancers (Yang et al., 2018). Blocking Pfkfb3 reduces proliferation and migration of endothelial cells in inflammatory diseases to prevent pathological angiogenesis (Schoors et al., 2014; Cruys et al., 2016). In addition, inhibition of PFKFB3 was shown to put proliferating endothelial cells in a quiescent state to maintain phenotypic homeostasis. In tumor microvessels, PFKFB3 inhibition via 3PO reduces the expression of EC adhesion molecules by inhibiting the NF-κB signaling pathway (Cantelmo et al., 2016). Glycolysis is increased in vascular ECs in areas prone to atherosclerotic lesions and in ECs exposed to blood flow disturbances *in vitro*, accompanied by enhanced inflammatory signaling (Yang et al., 2018). Downregulation of glycolytic enzymes in ECs decreases NF-κB activity and inflammation (Wang et al., 2019). The PFKFB3-associated and endothelial inflammatory diseases is summarized in Table 1.

**TABLE 1:** Endothelial inflammatory diseases associated with altered PFKFB3 expression.

Inflammatory Diseases	PFKFB3 expression	Pathomechanisms	Ref.
Pulmonary hypertension	↑	Hypertensive conditions increase expression of PFKFB3, leading to increased expression levels of endothelial cell growth factor and pro-inflammatory factor	Cao et al., 2019
Sepsis	↑	Increased PFKFB3 in septic lung upregulates expression levels of adhesion molecules and enhances leukocyte adhesion	Wang et al., 2019
Preeclampsia	↓	Reduced MALAT1 expression downregulates PFKFB3 expression, decreasing glycolytic activity, consequentlyresulting indysangiogenesis	Li et al., 2021
Ocular neovascular diseases	↑	Elevated expression levels of PFKFB3 increased cell endothelial cell proliferation, migration, and angiogenesis	Feng et al., 2021
Diabetic kidney disease	↑	IGFBP5 increases expression levels of EGR1. The latter binds to the PFKFB3 promoter, inducing PFKFB3 expression leading to enhanced glycolysis, cell proliferation and migration	Song et al., 2022

Pulmonary hypertension is a serious lung disease characterized by remodeling of small pulmonary vessels, associated with adverse vascular remodeling, including pulmonary vessel obstruction, hardening, and vasoconstriction (Mocumbi et al., 2024), resulting in a gradual increase in pulmonary vascular resistance, ultimately leading to right ventricular failure and death. The pathological changes in pulmonary hypertension include increased proliferation and resistance to apoptosis of pulmonary artery endothelial and smooth muscle cells, increased production and accumulation of extracellular matrix (Cao et al., 2019), increased local expression of proinflammatory cytokines and chemokines, and leukocyte infiltration into the perivascular region of the lung (Hu et al., 2020). The important pathogenic mechanism of pulmonary hypertension is abnormal metabolism, especially aerobic glycolysis or Warburg effect (Cao et al., 2019). PFKFB3-mediated glycolysis in ECs can increase the production of growth factors and proinflammatory cytokines in lung ECs (Cao et al., 2019). Through autocrine and paracrine pathways, these factors promote inflammatory response and pulmonary artery smooth muscle cells proliferation of lung ECs in animal models of pulmonary hypertension. It has been suggested that the ability of PFKFB3 to promote endothelial dysfunction is caused by increased expression of hypoxia-induced factor (HIF), which is stabilized by higher levels of glycolytic metabolites (Cao et al., 2019). Studies on transgenic mice have shown that hypoxia-inducible faction-2α (HIF-2α, HIF2A) and prolyl hydroxylase domain protein 2 (PHD2) are critical for Pulmonary hypertension development (Tang et al., 2018; Dai et al., 2016). These molecules play an important role in cell energy production, and endothelial cells HIF-2α and PHD2 are likely to promote PH by altering endothelial metabolism. Cao et al. (2019) revealed the key role of glycolytic regulatory factor PFKFB3 in the development of Pulmonary hypertension in the rodent Pulmonary hypertension model, and found that the inhibition of cellular PFKFB3 reduced the level of HIF2A, resulting in reduced production of growth factors, pro-inflammatory cytokines and chemokines, inhibition of adhesion molecules and attenuation of Pulmonary hypertension.

Sepsis is caused by infection or injury that adversely affects microvascular metabolism and immune-inflammatory homeostasis (Deutschman and Tracey, 2014; Angus and van der Poll, 2013; Xiao et al., 2023). Excessive inflammatory responses can cause severe cellular and tissue damages and organ dysfunction, such as acute lung injury (Imam et al., 2015; Shen et al., 2023), where ECs in the lining of blood vessels mediate vascular tone and the balance of proinflammatory and anti-inflammatory responses (Galley and Webster, 2004). Endothelial activation and dysfunction, characterized by upregulation of adhesion molecules and increased leukocyte adhesion, are characteristics of sepsis and can cause multiple organ dysfunctions (Aird, 2003). Studies have found that the expression of glycolytic regulatory factor PFKFB3 is significantly upregulated in sepsis lung, especially in lung endothelial cells (Wang et al., 2019), and experimental data show that endothelium-specific PFKFB3-mediated endothelial inflammation, including leukocyte infiltration mediated by increased expression of adhesion molecules, leads to the development of acute lung injury. In addition, NF-κB is highly activated in patients with sepsis and plays a key role in acute lung injury. Therefore, in a mouse model of LPS-induced sepsis, endothelium-specific PFKFB3 deficiency or 3-(3-pyridyl)-1-(4-pyridyl) -2-propenyl-1-one (3PO) inhibition of PFKFB3 significantly reduced pulmonary edema and improved survival. This is mainly due to the inhibition of the activity of NF-κB signaling pathway, which can inhibit the expression of LPS-induced inflammatory cytokines and adhesion molecules, reduce neutrophil influx, and thus reduce leukocyte infiltration and endothelial permeability (Wang et al., 2019). In addition, Zinc fingers and homeoboxes 2 (Zhx2) increases glycolytic metabolism in macrophages in a PFKFB3-dependent manner, resulting in enhanced inflammation during sepsis, whereas bone marrow-specific Zhx2 null mice are more resistant to sepsis induced by either LPS or cecal ligation and puncture (Wang et al., 2020).

Preeclampsia is a heterogeneous disease, affecting 3%–5% of pregnant women (Mol et al., 2016). Preeclampsia includes two major subtypes, i.e., early-onset preeclampsia and late-onset preeclampsia. Proper formation of blood vessels in the placenta ensures an adequate supply of oxygenated and nutritious blood to the foetus, a prerequisite for a successful pregnancy (Harris et al., 2019). Abnormal fetal vascular development can lead to placental vascular disease and affect fetal intrauterine development. ECs are a key determinant in angiogenesis in both healthy and diseased conditions (De Bock et al., 2013a; Breier et al., 2017). Vascular EC dysfunction reduces placental angiogenesis and promotes preeclampsia (Roberts et al., 1989; Chaiworapongsa et al., 2014). Expression levels of PFKFB3 and metastasis-associated lung adenocarcinoma transcript-1 (MALAT1) are reduced in placental tissues of early-onset preeclampsia patients (Li et al., 2021). *In vitro*, MALAT1 functions as a ceRNA of miR-26a and miR-26b and inhibits the expression of PFKFB3 in ECs. Knocking down MALAT1 can inhibit the expression of PFKFB3, thereby reducing glycolytic activity and ultimately leading to abnormal angiogenesis in early-onset preeclampsia. Knockdown of MALAT1 inhibits EC proliferation by inducing cell cycle arrest in the G0/G1 phase, and reduces EC motility by inhibiting cell process formation in a PFKFB3-dependent manner. Overexpression of PFKFB3 ameliorates angiogenesis caused by decreased MALAT1 expression in ECs. Collectively, the MALAT1/miR-26/PFKFB3 axis regulates EC angiogenesis by regulating glycolysis and plays a key role in the pathogenesis of early-onset preeclampsia (Li et al., 2021).

Ocular neovascular diseases, specifically posterior segment neovascularization, are the main cause for vision impairment and irreversible blindness in developed and developing countries (Campochiaro, 2013; Zhang and Ma, 2007). Increased EC activation, such as abnormal proliferation and migration stimulated by hypoxia and ischemia, is a major cause of vascular germination (Carmeliet and Jain, 2011). Hypoxia promotes YAP expression and nuclear translocation in human umbilical vein ECs. YAP acts as a transcriptional coactivator and binds to the PFKFB3 promoter with TEAD1, thereby increasing the expression of PFKFB3 (Feng et al., 2021). Silencing of YAP inhibits hypoxia-enhanced endodermal glycolysis and activation (such as cell proliferation and blood vessel germination), which all can be reversed by overexpression of PFKFB3. Additionally, intravitreal injection of either YAP or PFKFB3 siRNA significantly inhibits the neovasculargrowth (Feng et al., 2021). This line of evidence provides new insights into the targeting of the YAP/PFKFB3 axis for the treatment of ocular neovascularization treatment.

Diabetic kidney disease is a chronic, progressive disease (Koye et al., 2018). Diabetes-induced glomerular EC dysfunction is associated with the acquisition of pro-inflammatory and pre-thrombotic phenotypes, which facilitate the adhesion and infiltration of immune cells, and damage the integrity of fenestrated ECs, and increased endothelium-to-mesenchymal transformation (Zeng et al., 2022; Song et al., 2022; Fu et al., 2015). Activation of endothelial inflammatory phenotypes and synthesis of pro-inflammatory factors are all energy-dependent processes, and metabolic pathways in ECs can independently reprogram EC phenotypes (De Bock et al., 2013a; De Bock et al., 2013b). Glucose-induced expression of EGR1 is associated with the pathological changes of diabetic retinopathy (Ao et al., 2019). IGFBP5 can increase expression of EGR1, while the binding of EGR1 to the PFKFB3 promoter induces the expression of PFKFB3, accompanied by enhanced glycolysis (Song et al., 2022). Several studies have shown that PFKFB3-driven glycolysis in ECs increases inflammation. EC activation is a hallmark of diabetes, and glomerular EC dysfunction plays a key role in the development and progression of diabetic kidney disease (Yu et al., 2023). Diabetic nephropathy shows elevated expression levels of PFKFB3, which promotes glycolysis and inflammatory responses of ECs. Inhibition of PFKFB3 not only reverses the increase in glycolytic activity, but also inhibits endothelial inflammation and monocyte migration (Immanuel and Yun, 2023). Taken together, this line of evidence suggests that the glycolytic pathway of vascular ECs can serve as target for the treatment of angiogenesis-related diseases. This speculation is supported by that inhibition of PFKFB3 activity with 3PO inhibits angiogenesis in murine models of psoriasis and colitis (Schoors et al., 2014). Interestingly, in animal models of macular degeneration and retinopathy, which both are caused by blood vessel hyperproliferation, targeting PFKFB3 can also enhance the effect of VEGF signaling blocking on angiogenesis (Zhou et al., 2021).

## Summary

The pathogenic roles of PFKFB3 in inflammatory diseases include increasing glucose glycolysis, enhancing angiogenesis and stimulating proliferation of ECs. PFKFB3 can be a promising therapeutic target. Undoubtedly, development of an effective and safe PFKFB3 inhibitor can benefit some inflammatory disorders. Although PFKFB3 inhibitors 3PO and 7, 8-dihydroxy-3 -(4-hydroxyphenyl) -Chromen4-one (YN1) decreased angiogenic germination and HUVECs glycolysis rates both *in vitro* and *in vivo* (Schoors et al., 2014). However, it is worth noting that 3PO has a narrow therapeutic index, and the dose mediating vascular normalization and improving barrier function is only slightly lower than the dose causing toxicity and barrier destruction (Conradi et al., 2017). The maximum inhibitory effect of 3PO on glycolysis (Schoors et al., 2014) depends on high doses of 3PO (40 μM), which also has great potential to disrupt endothelial connections (Conradi et al., 2017). In addition, it was reported that 3PO is inactive in the PFKFB3 kinase assay, and there was no crystal structure to confirm 3PO binding to PFKFB3 kinase (Boyd et al., 2015). The anti-glycolytic activity of 3PO depends on its ability to interfere with the acidification of the intracellular environment, rather than directly binding to PFKFB3 (Emini et al., 2020). Therefore, there is still a long way to go to develop PFKFB3 inhibitors for clinical application.

## Abbreviations

PFKFB3, phosphofructokinase-2/fructose 2,6-bisphosphatase 3; PFK-1, phosphofructokinase-1; PFKFB, PFK-2/FBPase; 3PO, 3-(3-Pyridinyl)-1-(4-Pyridinyl)-2-Propen-1-One; F6P, fructose 6-phosphate; F1, 6P2, fructose 1, 6-bisphosphate; F2, 6P2, fructose 2, 6-bisphosphate; PFK-1, fructose-6-phosphate 1-kinase; PI, inorganic phosphate; VEGF, Vascular endothelial growth factor; ECs, Endothelial cells; Nrf2, nuclear factor (erythrocyte derived 2) -like2; LPS, lipopolysaccharides.
